# SNRPB2: a prognostic biomarker and oncogenic driver in esophageal cancer via β-catenin/c-Myc signaling

**DOI:** 10.3389/fonc.2025.1536473

**Published:** 2025-04-15

**Authors:** Jiaqian Bao, Xiong Tian, Yixiao Pan, Yiqing Guo, Zhenyu Yang, Meifu Gan, Jingmin Zheng

**Affiliations:** ^1^ Department of Public Research Platform, Taizhou Hospital of Zhejiang Province Affiliated to Wenzhou Medical University, Linhai, China; ^2^ Key Laboratory of Minimally Invasive Techniques & Rapid Rehabilitation of Digestive System Tumor of Zhejiang Province, Taizhou Hospital of Zhejiang Province Affiliated to Wenzhou Medical University, Linhai, China; ^3^ Department of Pathology, Taizhou Hospital of Zhejiang Province Affiliated to Wenzhou Medical University, Linhai, China

**Keywords:** SNRPB2, esophageal cancer, prognostic marker, EMT, β-catenin/c-Myc signaling pathway

## Abstract

**Background:**

The SNRPB2 gene encodes Small Nuclear Ribonucleoprotein Polypeptide B2, a crucial component involved in RNA splicing processes. While SNRPB2 dysregulation has been observed in various cancers, its role in esophageal cancer (ESCA) remains unclear.

**Methods:**

The mRNA level of SNRPB2 in ESCA was evaluated in combination with TCGA, GTEX, and GEO databases. The prognostic value of SNRPB2 was assessed using Kaplan-Meier analysis. Immunohistochemistry (IHC) was employed to confirm the expression of the SNRPB2 protein in tumor tissues from clinical samples. The biological functions of SNRPB2 were assessed *in vitro* cell assay and *in vivo* tumor models. The molecular mechanisms were determined by correlation and gene set enrichment analysis. Western blot experiments validated involvement in signaling pathways.

**Results:**

Our findings unveiled that SNRPB2 was upregulated at both mRNA and protein levels in ESCA, which was associated with the pathological progression of the disease. Additionally, SNRPB2 served as a robust prognostic biomarker, implicated in driving oncogenic functions in ESCA. It facilitated cell proliferation, migration, and invasion, transitioned the cell cycle, and inhibited apoptosis. Mechanistically, SNRPB2 activated genes associated with the β-catenin/c-Myc signaling pathway, such as β-catenin, c-Myc, CCNA2, CCNB1, CDK1, and CDK2. This activation also regulated the epithelial-to-mesenchymal transition (EMT), thereby facilitating the progression of ESCA.

**Conclusion:**

Our findings demonstrate that SNRPB2 contributes to ESCA progression by regulating the β-catenin/c-Myc axis, suggesting its potential as a prognostic biomarker and therapeutic target for ESCA patients.

## Introduction

Cancer is a leading cause of death globally, imposing a significant economic strain on public health systems (1, 2). Esophageal cancer (ESCA) is a common and severe malignant tumor that primarily affects the upper gastrointestinal tract. Its incidence ranks seventh worldwide, while its mortality ranks sixth (3). Due to the subtle early clinical symptoms, most patients are usually diagnosed at intermediate or advanced stages, often characterized by distant metastasis (4). Despite advances in clinical diagnosis and treatment, particularly with targeted therapies involving immune checkpoint inhibitors, optimism regarding overall treatment outcomes for ESCA remains limited (5–7). The five-year survival rate for ESCA remains remarkably low, ranging from approximately 15% to 25% (8). In recent years, Tumor biomarkers have been widely used in diagnosing and treating tumors (9, 10).Such as human epidermal growth factor receptor 2 (HER2), epidermal growth factor receptor (EGFR), vascular endothelial growth factor receptor (VEGFR), and programmed death-ligand 1 (PD-L1) have been utilized in the diagnosis and treatment of ESCA (11–14). However, it is noteworthy that only a limited proportion of patients derive clinical benefit from these targeted therapies. Therefore, identifying more suitable and effective therapeutic targets holds significant potential for advancing the treatment of ESCA.

Computational bioinformatics has transformed cancer diagnosis and treatment by analyzing RNA, proteins, and metabolites to identify biomarkers for early detection, therapy monitoring, and personalized care (15–17). Guozhong Jiang et al. determined the molecular subtypes of esophageal squamous cell carcinoma through bioinformatics analysis (18). Rao D et al. through proteomic and bioinformatics analysis identified exosome-derived CD45 as a promising biomarker for ESCA diagnosis (19). Nguyen-Kieu Viet-Nhi et al. comprehensive multi-omics bioinformatics analysis identifies IFI6 as a biomarker and treatment target for predicting outcomes in ESCA (20).

The spliceosome is an intricate RNA-protein complex whose primary role is to excise intronic regions from eukaryotic gene sequences through splicing reactions. This process facilitates the joining of exonic sections, forming mature mRNA molecules (21). It is composed of several small nuclear ribonucleoproteins (snRNPs), which are essential for the process of RNA splicing. Each snRNP is made up of uridine-rich small nuclear RNA (U snRNA) and various Sm proteins, including SNRPB/B’, D1, D2, D3, E, F, and G. Additionally, an assortment of associated proteins is present in varying quantities (22). Deviations in the fundamental components of the spliceosome are often linked to various phenotypic expressions and pathological conditions (23). SNRPB2, recognized as U2B, was first identified as a key element in the assembly and functionality of spliceosomes (24). A growing body of evidence suggests a close association between SNRPB2 and tumor progression. Deng et al. found that multiple myeloma patients with low SNRPB2 expression had better prognoses, and inhibiting SNRPB2 increased the sensitivity of multiple myeloma to treatment with ixazomib (25). Yue Luo et al. reported that SNRPB2 was upregulated in hepatocellular carcinoma and acted as a poor prognostic indicator (26). The expression of SNRPB2 was associated with poor prognosis and promoted triple-negative breast cancer progression (27). CD28 within cancer cells also enhances PD-L1 expression by stabilizing CD274 mRNA through its interaction with SNRPB2, driving immune evasion in triple-negative breast cancer (28). However, the expression of SNRPB2 in ESCA remains unclear, and its clinical significance necessitates further investigation.

In this study, we combined bioinformatics analysis with experimental validation to reveal that SNRPB2 is overexpressed in ESCA, which is closely linked to the disease prognosis. Immunohistochemical analysis of clinical samples revealed a significant correlation between high SNRPB2 expression and the pathological stage of ESCA. Additional investigations, including both *in vivo* and *in vitro* studies, indicated that SNRPB2 facilitates ESCA progression by modulating the β-catenin/c-Myc signaling pathway and promoting epithelial-mesenchymal transition (EMT). Thus, SNRPB2 may serve as a promising novel biomarker for prognosis and therapy in ESCA.

## Materials and methods

### Data collection and bioinformatic analysis

Pan-cancer expression profiles and related clinical data were obtained from the UCSC Xena database (https://xenabrowser.net/datapages/), including data from the Genotype-Tissue Expression (GTEx) and the Cancer Genome Atlas (TCGA) project. At the same time, obtained three expression profiles of SNRPB2 (GSE20347, GSE53625, and GSE23400) from the Gene Expression Omnibus (GEO) database (https://www.ncbi.nlm.nih.gov/). The “ggplot2” package in R formed box plots illustrating the expression distribution. Using the R package ‘survival’, we conducted Kaplan-Meier analysis to investigate the impact of SNRPB2 on the prognosis of ESCA. According to the median of SNRPB2 expression, the patient was divided into a highly expressed group and a lowly expressed group.

### Clinical specimens

A retrospective collection of 162 paraffin-embedded ESCA tissue samples was conducted between May 2004 and 2018 at the Pathology Department of Taizhou Hospital, Affiliated with Wenzhou Medical University(Linhai, China). All tissue samples underwent clinical pathological analysis and were diagnosed as ESCA. **Table 1** contains detailed clinical data and specimen information. This study was conducted based on written informed consent from all patients, ensuring ethical standards and the right to informed participation, and received approval from the Ethics Committee of Taizhou Hospital in Zhejiang Province (No.K20210618).

**Table 1:** Association between clinical features and SNRPB2 expression of ESCA patients.

Variable	Low expression(n=44)	High expression(n=118)	p-value
Age(year)			
<63	23	58	
≥63	21	60	0.72
Gender			
Male	26	74	
Female	18	44	0.67
Tumor length (cm)			
≤3	21	43	
>3	23	75	0.21
T stage			
T1-2	19	47	
T3-4	25	71	0.77
N stage			
N0	22	63	
N1-3	22	55	0.70
TNM stage			
Stage I/II	29	45	
Stage III/IV	15	73	**0.002**
Smoking			
Yes	26	63	
No	18	55	0.60
Drinking			
Yes	31	85	
No	13	33	0.85
Diabetes			
Yes	40	102	
No	4	16	0.60
Hypertension			
Yes	33	80	
No	11	38	0.44

### Immunohistochemistry staining

IHC staining was performed following the established protocol (29). The 4 μm sections were obtained by cutting the paraffin-embedded tissue blocks and then attached them to glass slides. Following deparaffinization, rehydration, and microwave antigen retrieval, the slides were incubated overnight at 4°C with antibodies SNRPB2 (Thermo Fisher, PA5-84991) and Ki-67 (Roche, Cat. No. 790-4286). After a 30-minute incubation with a secondary antibody at room temperature, the slides underwent staining using a DAB substrate. Subsequently, counterstaining with hematoxylin was performed. The final staining score was determined based on staining intensity, which ranged from 0 for negative, 1+ for weak, 2+ for moderate, 3+ for strong. Scores of 0 to 1 were considered indicative of low expression, while scores between 2 and 3 were classified as high expression.

### Cell culture

The Eca109 and Kyse150 cell lines were sourced from the Cell Bank of the Chinese Academy of Sciences in Shanghai, China. These cell lines were maintained in RPMI 1640 medium (Meilun Biology, Dalian, China) supplemented with 10% fetal bovine serum (FBS) and incubated at 37°C in a 5% CO2 atmosphere. To investigate whether SNRPB2 regulates the expression of c-Myc through β-catenin, SNRPB2-knockdown cells and Sh-NC cells were treated with the β-catenin agonist SKL2001(MCE HY-101085) at 20 µmol/L for 24 h. SNRPB2-overexpressed cells were treated with β-catenin inhibitor XAV-939 (MCE HY-15147) at 20 µmol/L for 24 h.

### Lentiviral vector transduction

In order to obtain stable cell lines, three SNRPB2 gene shRNA interference sequences and overexpression sequences were respectively cloned into pLVshRNA-EGFP(2A) puro and pCDH-CMV-MCS-EF1 vectors. These constructs were co-transfected with corresponding helper plasmids into 293T cells. After 48 and 72 hours of transfection, collected the supernatant and filtered through a 0.45 μm filter for further purification. Then used the filtered supernatant was mixed with 4μg/ml Polybrene to infect ESCA cells. Infected cells were selected by treating them with puromycin at a concentration of 2μg/ml for 3 days, and subsequent experiments were conducted.

### RNA isolation and qPCR detection

Total RNA was isolated from Eca109 and Kyse150 cells using TRIzol reagent, with the extraction process conducted in strict accordance with the manufacturer’s instructions. Total RNA content and concentration was quantified using a Nucleic Acid Quantification Analyzer (Nanodrop, USA). Subsequently, the RNA was converted into cDNA using a reverse transcription PCR kit under specific conditions: incubation at 37°C for 30 minutes, denaturation at 85°C for 15 seconds, and storage at 4°C indefinitely. The expression level of SNRPB2 was determined using qPCR with 18s rRNA serving as the internal reference. The relative expression levels of SNRPB2 were calculated using the formula 2^(-ΔΔCT). The primers for qPCR employed in this study were as follows:

SNRPB2 forward primer: 5’-TGAGACAGCTACAAGGATTTCCA-3’
SNRPB2 reverse primer: 5’-CAGCAAAAGTTCCACGCATTT-3’
18s forward primer: 5’-TTTCTCGATTCCGTGGGTGG-3’
18s reverse primer: 5’-AGCATGCCAGAGTCTCGTTC-3’

### Western blot

ESCA cells were subjected to protein extraction using RIPA lysis buffer. The concentration of the extracted protein was then measured with a BCA protein assay kit (Beyotime, Jiangmen, China). Subsequently, twenty micrograms of protein were loaded onto a polyacrylamide gel for electrophoretic separation, and the separated proteins were transferred to polyvinylidene fluoride (PVDF) membranes. For immunodetection purposes, primary antibodies including anti-SNRPB2 (1:1000; PA5-84991,thermo),anti-Vimentin(1:1000;5741,CST),anti-N-cadherin(1:1000;14215, CST), anti-E-cadherin(1:1000;GTX100443,Genetex),anti-ZO1(1:1000;GTX636491,G enetex),anti-β-catenin(1:1000,GTX633010, Genetex), anti-c-Myc(1:1000,9402, CST),anti-CCNB1(1:1000;CY5378,abways), anti-CCNA2(1:2000;CY5837,abways), anti-CDK1(1:1000;CY5061,abways), anti-CDK2(1:1000;CY5020,abways), anti-β-actin (1:5000; YT0099, Immunoway), and anti-GAPDH(1:10000;60004-1-Ig,proteintech) were used in this study. The membranes were incubated overnight at 4°C with the primary antibodies, after which they were washed thoroughly. Secondary antibodies (at a dilution of 1:10000) were then added to the membranes. Finally, ECL was employed for color development and Image Quant LAS 500 (GE, USA) was used to detect the protein bands. The resulting images were quantified using the specialized software ImageJ.

### CCK-8 and EDU cell proliferation assay

Cell Counting Kit-8 (GLPBIO, GK10001) was utilized to evaluate cellular proliferation capacity. A total of 3000 cells were added to each well of a 96-well plate. After 24, 48, and 72 hours, each well was supplemented with a mixture of 100μL medium without serum and 10μL CCK-8 reagents. The plates were then incubated at 37°C for 1.5 hours, after which the absorbance at 450 nm was measured with a microplate reader to assess cellular metabolic activity. Furthermore, the EDU cell proliferation assay kit (Beyotime, C0085S) was used to evaluate cell proliferation. Briefly, ESCA cells were seeded in 6-well plates and allowed to reach optimal growth. EDU solution (10 mM) was added and incubated for 2 hours at 37°C. Following fixation with 4% paraformaldehyde, the cells were washed, permeabilized, and subjected to fluorescent dye staining. Flow cytometry analysis was performed to determine the number of proliferating cells that were labeled with EDU.

### Clone formation analysis

Cellular proliferation was evaluated through a cell colony formation assay. A total of 1,000 cells were plated in 60 mm dishes and cultured for seven days in a 5% CO2 environment until visible colonies formed. Subsequently, following fixation with 4% paraformaldehyde for 20 minutes, the formed colonies underwent staining using a 1% crystal violet solution for 15 minutes. After thorough drying, the colonies were meticulously enumerated to quantify their proliferative capacity.

### Cell wound scratch assay

The cells were cultured in a 6-well plate until they reached full confluency of 100%. To create uniform scratches on the cell monolayer, a 200μL pipette tip was carefully used. Following the scratching, serum-free medium was added to the wells. Subsequently, the cells were imaged using an inverted microscope after 12 to 24 hours to observe and quantify their Migration into the wounded area.

### Transwell invasion assays

For the cell invasion assay, the upper chamber was properly coated with extracellular matrix gel (Corning, 356234). After digesting the cells, a suspension in 100 µL of serum-free medium was prepared and then seeded into the upper chamber (Corning, Lowell, MA, USA). The lower chamber contained a 10% FBS culture medium, a chemoattractant to facilitate cell movement. Following a 24-hour incubation at 37°C, the cells in the lower chamber were fixed with 4% paraformaldehyde and subsequently stained with crystal violet. The number of invaded cells was quantified by counting in five randomly selected fields using an inverted microscope.

### Apoptosis and cell cycle assay

For cell cycle assay, Eca109 and Kyse150 cells were collected and subsequently fixed in ice-cold ethanol (70%) at a temperature of 4°C for 24 hours. Then, the cells were incubated with propidium iodide (10 mg/ml, Beyotime) for 30 min at room temperature in a dark place, and the distribution of the cell cycle distribution was analyzed using flow cytometry. For the apoptosis assay, cells were harvested and subjected to staining with Annexin V APC/7-AAD (Elabscience, China) according to the manufacturer’s instructions. The percentage of cell apoptosis was measured, and the results were analyzed by FlowJo V10 software.

### Animal experiments

BALB/c nude mice were obtained from Shanghai SLAC Laboratory Animal Co., Ltd (Shanghai, China). All procedures involving the mice were conducted in accordance with the guidelines and regulations set forth by the Animal Care Committee of Taizhou Hospital (No. tyz-2022130). To establish subcutaneous tumor models, male BALB/c nude mice aged 4 weeks were randomly assigned to four groups, each containing six mice. The groups included a blank group, a negative control (NC) group, and two experimental groups: SNRPB2-shRNA2 and SNRPB2-shRNA3. The digested cells were mixed with extracellular matrix gel in a 1:1 ratio and resuspended. Subsequently, each nude mouse was injected subcutaneously with 100 μL of the cell suspension-extracellular matrix gel mixture. A total of 1 × 10^6^ cells were inoculated into each mouse to ensure consistent cell dosage. Tumor images were captured after a 4-week incubation period, and its diameter was measured. Subsequently, the tumor masses were fixed, embedded, and subjected to immunohistochemical staining for further analysis.

### Statistical analysis

The presentation of all experimental data followed the mean ± standard deviation (SD) format. Statistical analyses were conducted using either GraphPad Prism (Version 8.0, CA, USA) or the R programming language. Each cell experiment was independently repeated three times. Comparisons between the two groups were assessed using either an unpaired two-tailed t-test or a chi-square test. A p-value of less than 0.05 was deemed statistically significant.

## Results

### SNRPB2 was upregulated expression in ESCA and serves as a reliable prognostic biomarker

**Figure 1:**
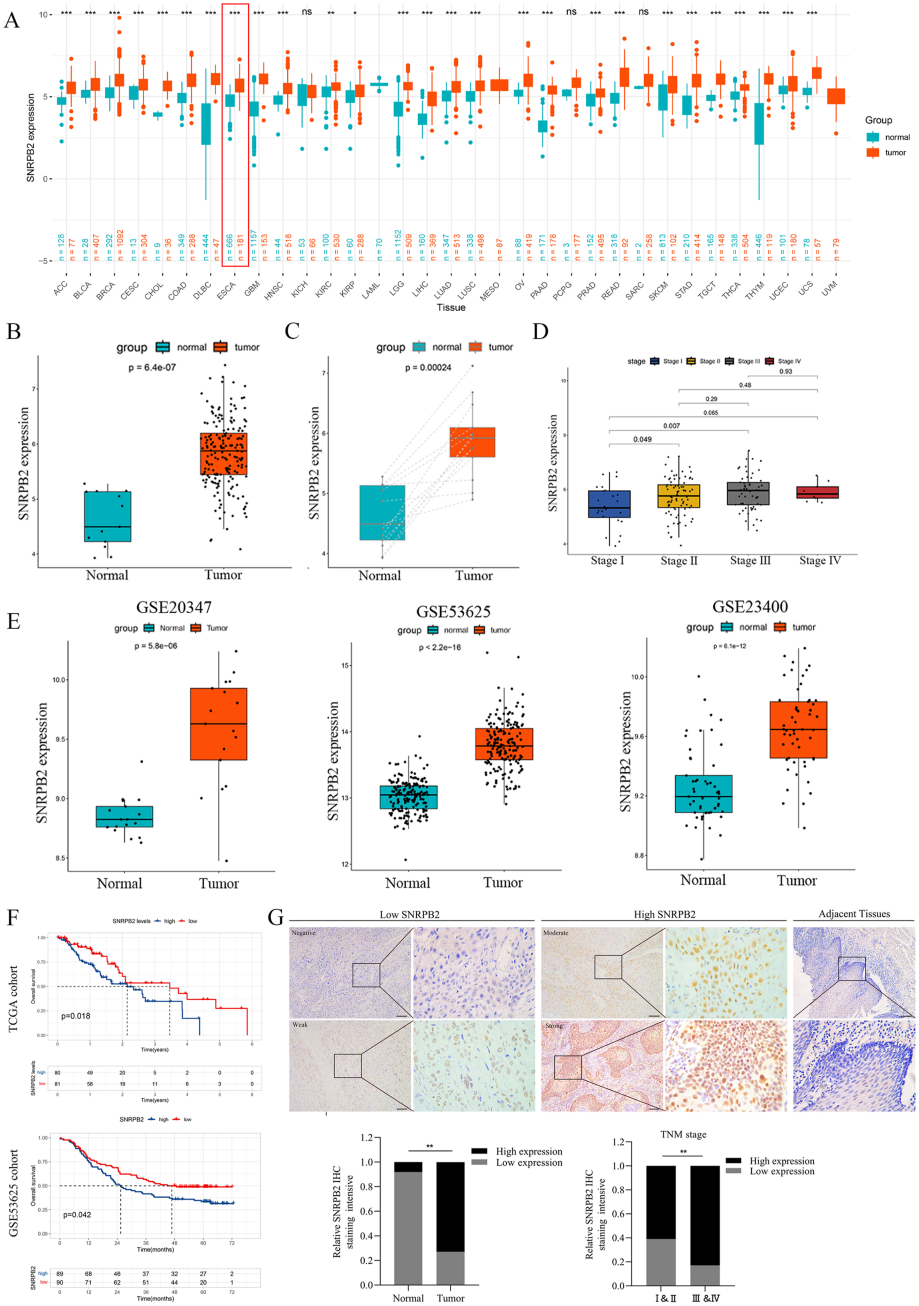
SNRPB2 upregulation in ESCA and its potential as a prognostic biomarker. **(A)** SNRPB2 expression analyzed by TCGA and GETx datasets. *p < 0.05, **p < 0.01, ***p < 0.001, ns, no significant differences. **(B)** The expression level of SNRPB2 in ESCA tissues was upregulated compared with that of normal tissues in the TCGA database. **(C)** The expression level of SNRPB2 in ESCA tissues was upregulated compared with that of paired samples in the TCGA database. **(D)** The expression level of SNRPB2 was associated with clinical pathological staging. **(E)** Validation of SNRPB2 expression in ESCA tissues using the GEO database. **(F)** The prognostic significance of SNRPB2 mRNA in ESCA was explored by Kaplan–Meier analyses in TCGA and GSE53625 cohorts. **(G)** Representative SNRPB2 IHC staining in adjacent tissues and ESCA tissues. Comparison of IHC staining intensity between adjacent tissues and ESCA tissues. Comparison of staining intensity of SNRPB2 in different tumor stages. *P < 0.05, **P < 0.01. Scale bar:20µm.

The expression of SNRPB2 was analyzed across 33 tumor types through integration of samples from the GTEx and TCGA databases. Results revealed that SNRPB2 is upregulated in 27 tumor types, including ACC, BLCA, BRCA, CESC, CHOL, COAD, DLBC, ESCA, GBM, HNSC, KIRC, KIRP, LGG, LIHC, LUAD, LUSC, OV, PAAD, PRAD, READ, SKCM, STAD, TGCT, THCA, THYM, UCEC, and UCS (**Figure 1A**).

To further validate these findings, we analyzed data from the TCGA databases specifically for ESCA, which consistently demonstrated a significant increase in SNRPB2 expression levels in ESCA tissues compared to normal tissues (**Figure 1B**). This observation was corroborated when analyzing paired samples (**Figure 1C**). Additionally, our analysis revealed a positive correlation between SNRPB2 expression levels and the staging of ESCA (**Figure 1D**). Further validation was conducted using multiple ESCA tissues datasets within the GEO database, all of which showed elevated expression levels of SNRPB2 (**Figure 1E**). To evaluate the prognostic relevance of SNRPB2 in ESCA, Kaplan-Meier survival analysis revealed that high SNRPB2 expression in the TCGA and GSE53625 cohorts was associated with significantly poorer prognosis compared to low-expression groups (**Figure 1F**). These results indicated that SNRPB2 has the potential to be an important prognostic biomarker for ESCA.

### SNRPB2 was significantly upregulated in protein levels within ESCA clinical samples

IHC staining was conducted on ESCA tissues, and adjacent non-tumorous samples were matched to validate bioinformatic findings, systematically comparing SNRPB2 protein expression levels. The results showed a marked increase in SNRPB2 protein expression levels in ESCA tissues compared to adjacent non-cancerous tissues, confirming consistency with the bioinformatics data. Additionally, a significant correlation was observed between SNRPB2 protein expression and the staging of ESCA patients (**Figure 1G**).

### Knockdown of SNRPB2 suppressed the malignant phenotype of ESCA cells.

**Figure 2:**
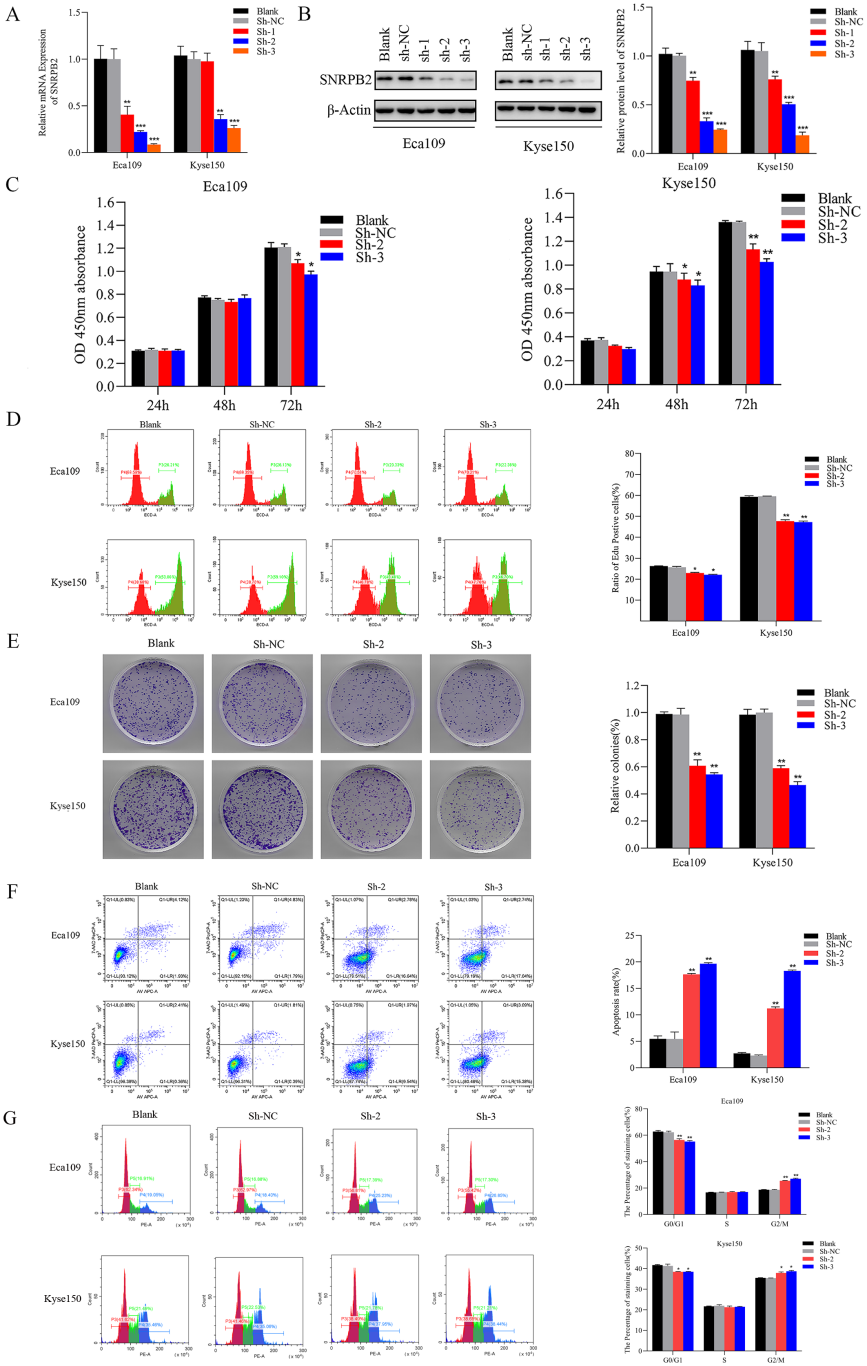
Knockdown of SNRPB2 suppressed the growth of ESCA cells. **(A)** Silencing efficiency of SNRPB2 in Eca109 and Kyse150 cells by qPCR. **(B)** Silencing efficiency of SNRPB2 in Eca109 and Kyse150 cells by Western blot. **(C–E)** CCK-8, EDU and colony formation assays showing the proliferation ability of Eca109 and Kyse150 cells. **(F,G)** Flow cytometry results showing the effect of SNRPB2 knockdown on apoptosis and cell cycle progression in Eca109 and Kyse150 cells. *P < 0.05, **P < 0.01, ***P < 0.001.

**Figure 3:**
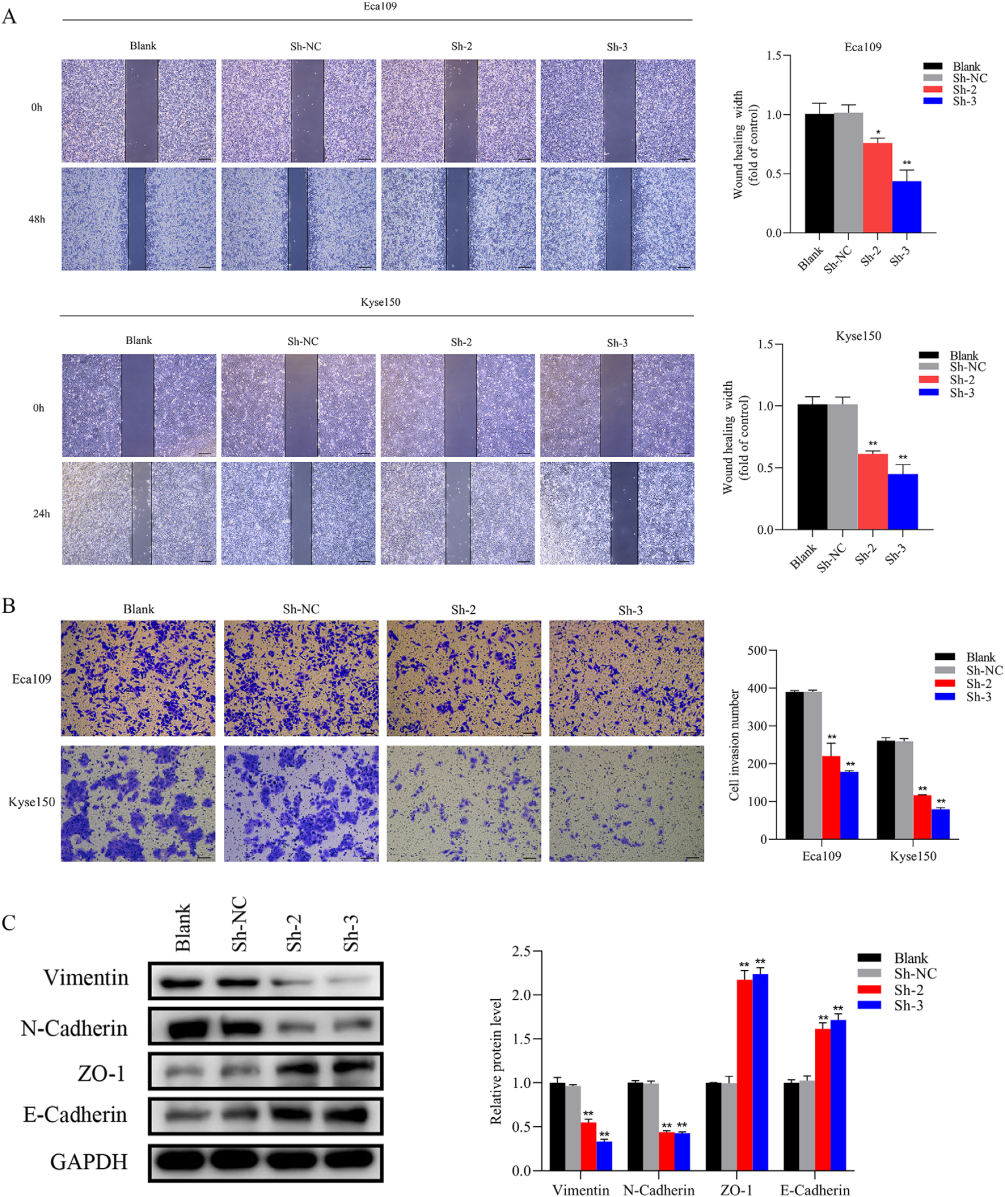
Knockdown of SNRPB2 suppressed the Migration and invasion through EMT. **(A)** Wound-healing assay to assess cell migration ability. **(B)** Transwell invasion assay to measure the cell invasion ability. **(C)** Western blot results showed changes in EMT. *P < 0.05, **P < 0.01. Scale bar:100 µm.

To interrogate SNRPB2 function in ESCA cells, stable knockdown cell lines were generated, with efficient depletion confirmed by qPCR (**Figure 2A**) and Western blot analysis (**Figure 2B**). Subsequently, two shRNA sequences exhibiting the highest interference efficiency were chosen for follow-up experiments. The results of the CCK-8 assay exhibited a remarkable decline in the proliferative capacity of Eca109 and Kyse150 cell lines following SNRPB2 knockdown, in comparison to the Sh-NC cells (**Figure 2C**). Further assessment using the EDU assay confirmed that SNRPB2 knockdown significantly suppressed the proliferative ability of Eca109 and Kyse150 cells (**Figure 2D**). Furthermore, quantitative analysis of the colony formation assay indicated a noticeable decrease in the number of colonies formed by SNRPB2-deficient cells compared to the Sh-NC cells (**Figure 2E**). Flow cytometry analysis revealed that SNRPB2 knockdown caused cell cycle arrest in the G2/M phase and promoted apoptosis (**Figures 2F, G**). In addition, we utilized scratch assays and Transwell invasion assays to assess the effects of SNRPB2 knockdown on tumor migration and invasion. The quantification of migrated and invaded cells revealed a substantial reduction in both *in vitro* Migration (**Figure 3A**) and invasion capability (**Figure 3B**) of ESCA cells following SNRPB2 knockdown.

### The overexpression of SNRPB2 facilitated the malignant phenotype of ESCA cells

**Figure 4:**
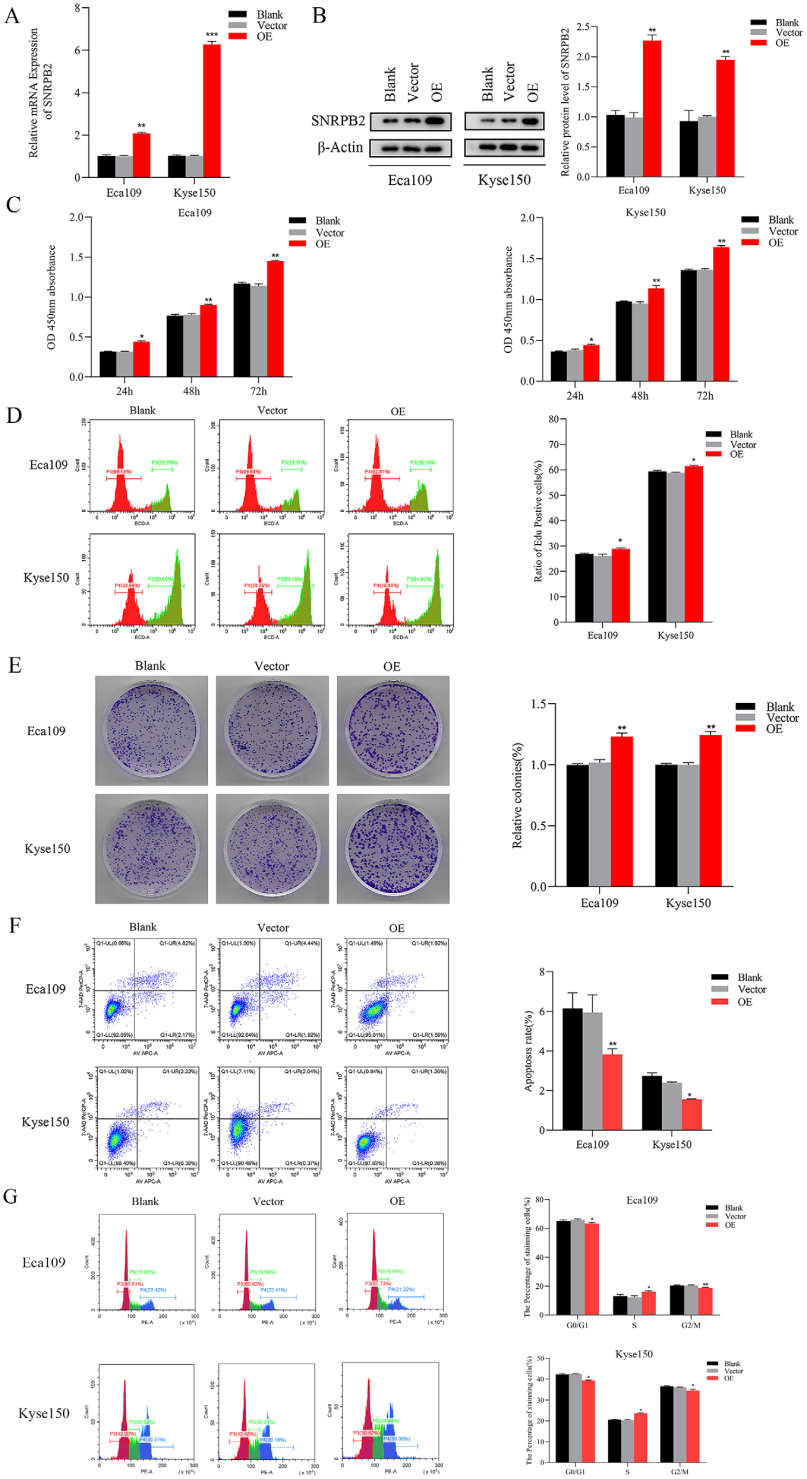
Overexpression of SNRPB2 promoted the growth of ESCA cells. **(A, B)** The efficiency of SNRPB2 overexpression was confirmed in Eca109 and Kyse150 cells through qPCR and Western blot. **(C–E)** Proliferation ability of Eca109 and Kyse150 cells upon SNRPB2 overexpression assessed by CCK-8, EDU, and colony formation assays. **(F, G)** Flow cytometry results showing the effect of SNRPB2 overexpression on apoptosis and cell cycle progression in Eca109 and Kyse150 cells. *P < 0.05, **P < 0.01.

**Figure 5:**
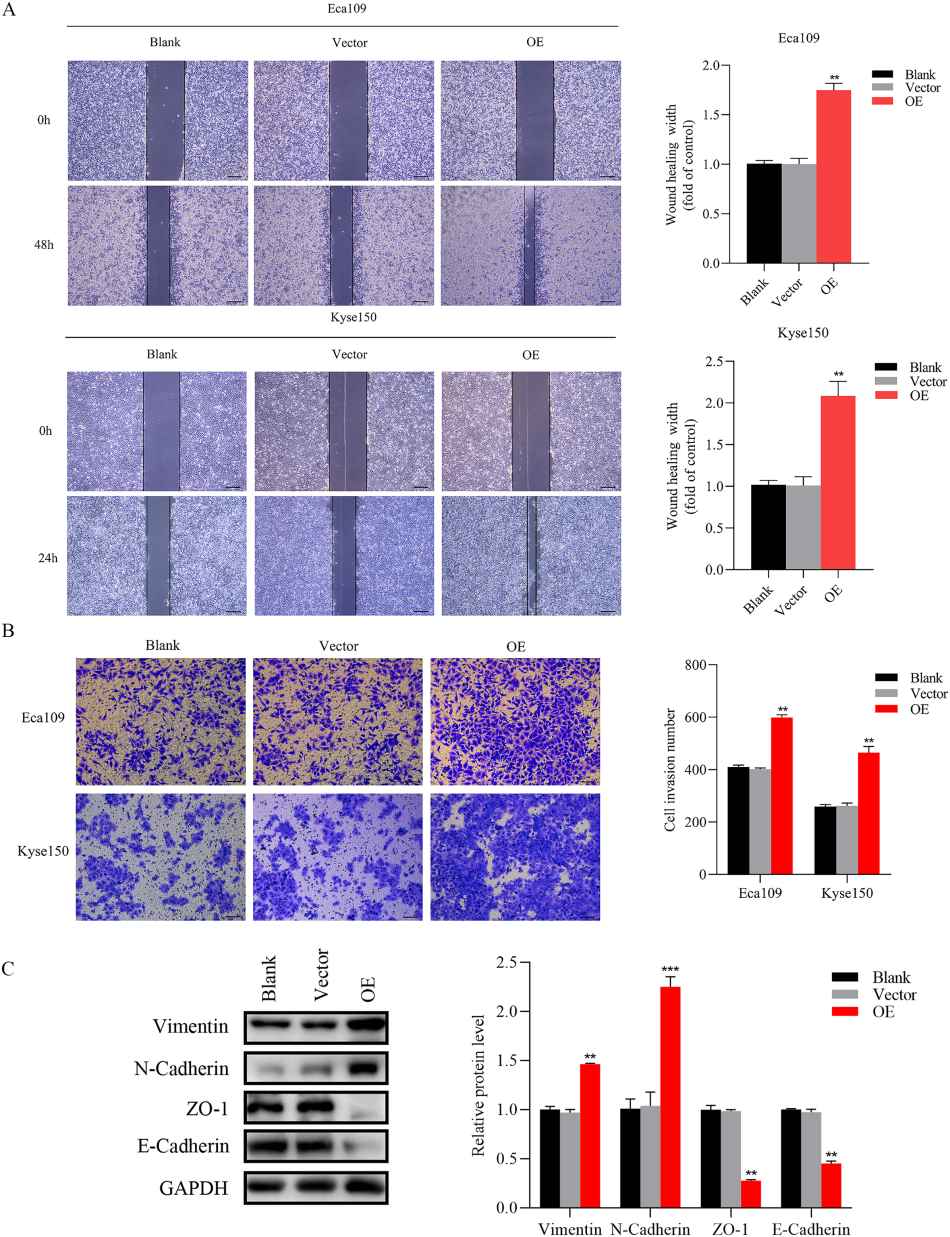
Overexpression of SNRPB2 enhanced Migration and invasion through EMT. **(A)** Wound-healing assay to assess cell migration ability. **(B)** Transwell invasion assay to measure the cell invasion ability. **(C)** Western blot results showed changes in EMT. **P < 0.01, ***P < 0.001. Scale bar:100 µm.

To further substantiate the involvement of SNRPB2 in ESCA progression, SNRPB2 overexpression experiments were conducted in ESCA cells. The efficacy of overexpression was validated through qPCR (**Figure 4A**) and Western blot (**Figure 4B**). Subsequently, comprehensive CCK-8 and EDU assays unequivocally demonstrated that SNRPB2 overexpression exerted a profound stimulatory effect on the proliferative capacity of ESCA cells (**Figures 4C, D**). Furthermore, the colony formation experiments illustrated the augmenting impact of SNRPB2 on the cellular proliferation process (**Figure 4E**). Flow cytometry analysis demonstrated that overexpression of SNRPB2 induced G2/M phase transition and attenuated cell apoptosis (**Figures 4F, G**). Strikingly, scratch assays convincingly revealed that SNRPB2 overexpression significantly expedited the migratory potential of ESCA cells (**Figure 5A**), while Transwell assays effectively substantiated its capability to enhance the invasive prowess of these cells (**Figure 5B**).

### SNRPB2 mediates the malignant proliferation and metastasis of ESCA cells through the EMT

The EMT signaling pathway exerts a pivotal role during the progression of tumor growth and the subsequent development of metastasis (30). Based on preliminary phenotypic analyses, we hypothesized that SNRPB2 may promote ESCA cells’ malignant proliferation and metastasis through the EMT process. As expected, Western blot analysis was performed to investigate the expression of key markers associated with epithelial and mesenchymal cells during the EMT process. The findings indicated that in the SNRPB2 knockdown group, the levels of mesenchymal markers, including Vimentin and N-cadherin, were significantly reduced, whereas the expression of epithelial markers, such as ZO-1 and E-cadherin, showed a notable increase compared to the Sh-NC group (**Figure 3C**). Conversely, in the SNRPB2 overexpression group, there was an upregulation of Vimentin and N-cadherin expression, accompanied by a suppression of ZO-1, E-cadherin expression (**Figure 5C**). These results highlight the crucial function of SNRPB2 in the *in vitro* proliferation and migration of ESCA cells, which is facilitated by its regulation of the EMT process.

### Knockdown of SNRPB2 arrested tumor growth in a nude subcutaneous xenograft mode

**Figure 6:**
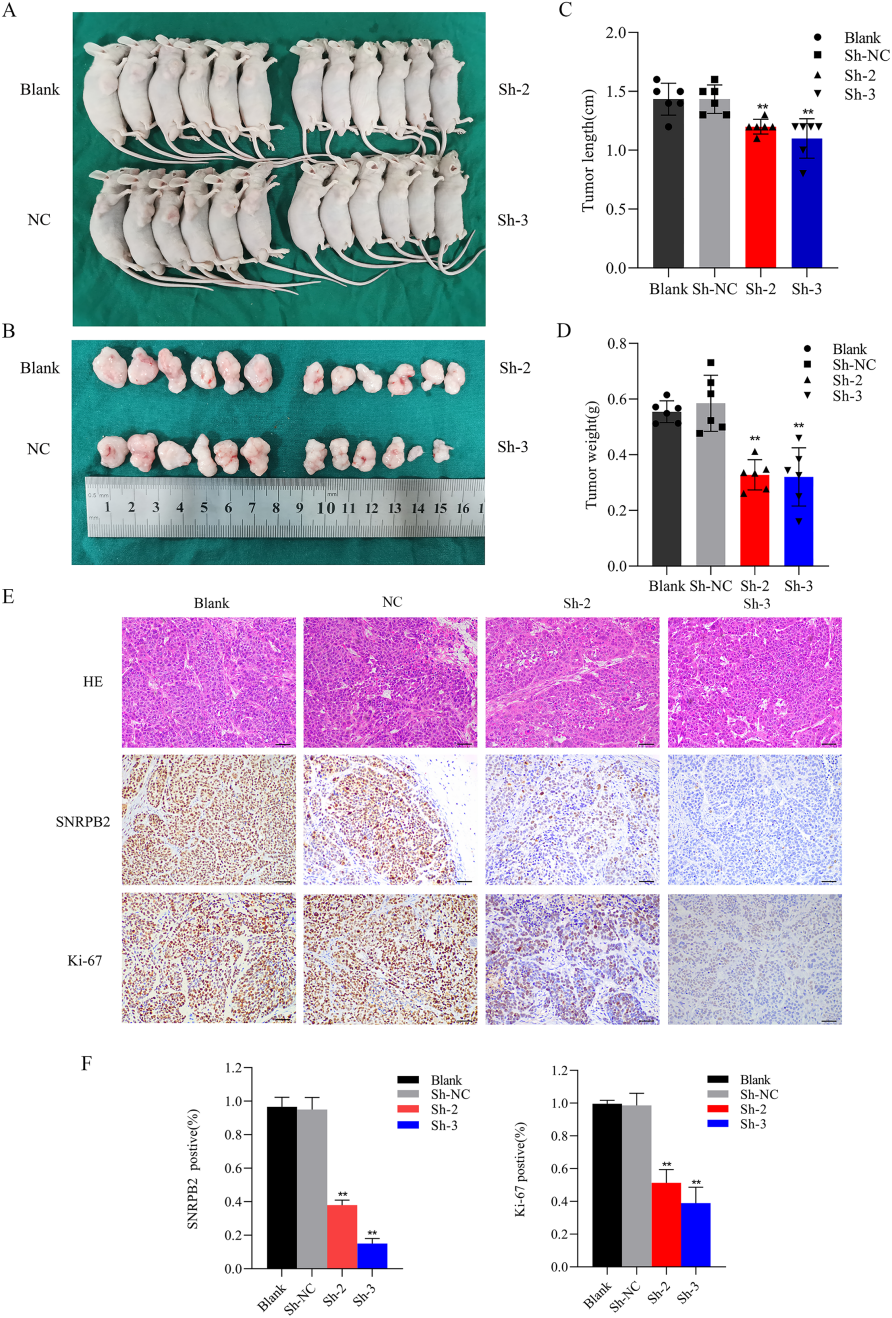
SNRPB2 knockdown inhibits ESCA cells growth *in vivo*. **(A, B)** Images depicting tumors formed in BALB/c nude mice following subcutaneous injection of SNRPB2-knockdown Eca109 cells. **(C)** The tumor volumes were measured after tumor resection. **(D)** The tumor weights were measured after tumor resection. **(E)** HE staining and immunohistochemical (IHC) staining of SNRPB2 and Ki-67 antibodies were performed on sections of subcutaneous tumors. **(F)** Expression analysis of SNRPB2 and Ki-67 across diverse tissue sections. **P < 0.01. Scale bar:20 µm.

In the subsequent study, we explored *in vivo* role of SNRPB2 in the progression of ESCA. Both SNRPB2 knockdown and control Eca109 cells were subcutaneously inoculated into nude mouse models. The results indicated that, in comparison to the Sh-NC group, the tumor volume in the shRNA-treated group was significantly smaller (p < 0.01), accompanied by a lighter tumor weight (p < 0.01), and a slower growth rate (**Figures 6A-D**). Histological examination using HE staining demonstrated a remarkable decrease in tumor cell density in the SNRPB2 knockdown group. Furthermore, immunohistochemical analysis showed consistent downregulation of SNRPB2 expression levels and Ki-67 expression in the shRNA-treated group. These findings further confirm that SNRPB2 knockdown can effectively inhibit the malignant proliferative capacity of ESCA cells *in vivo* (**Figures 6E, F**).

### SNRPB2 activates the β-catenin/c-Myc axis in the progression of ESCA

**Figure 7:**
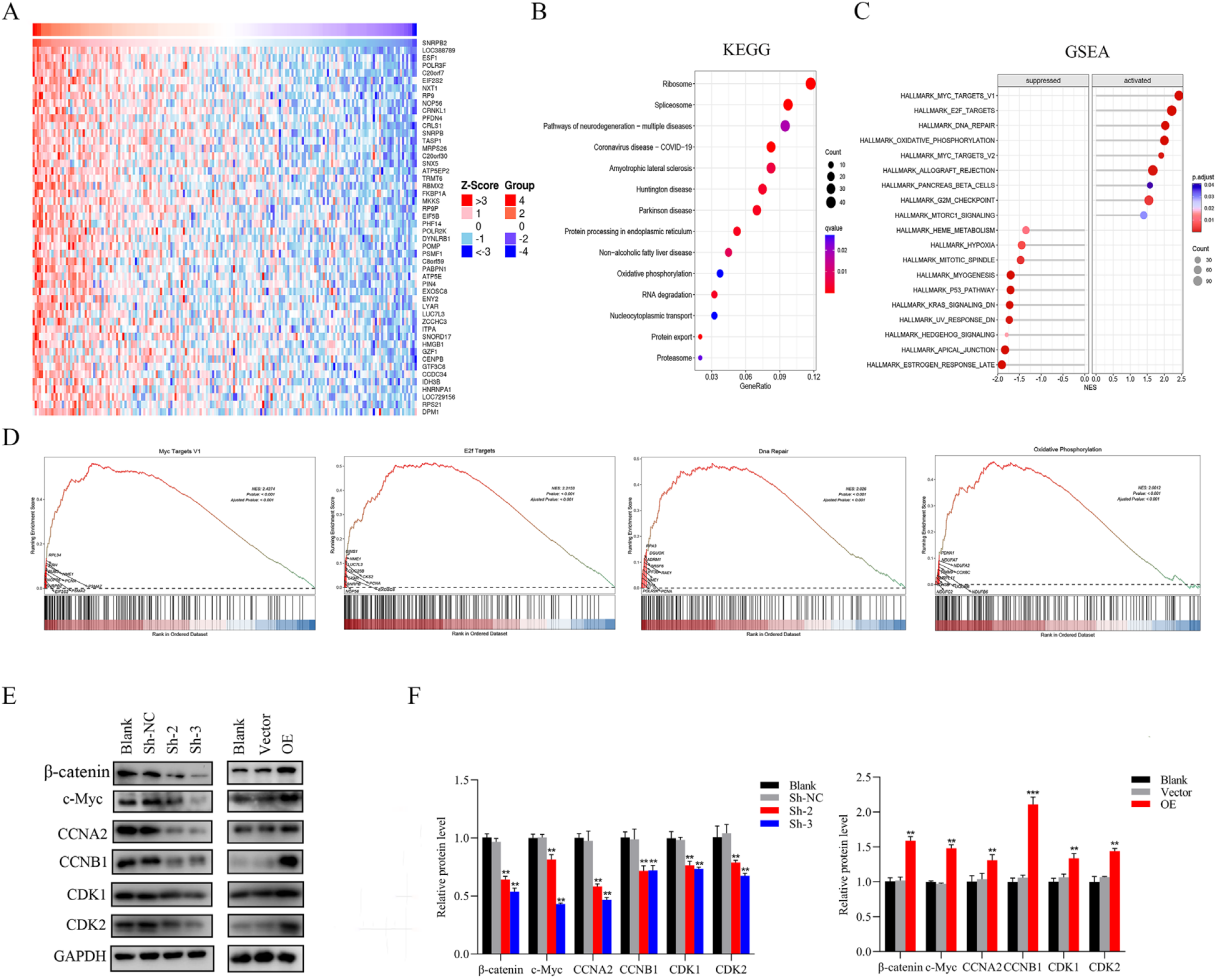
SNRPB2 regulates β-catenin/c-Myc signaling pathway in ESCA. **(A)** Heatmap showing the top 50 genes coexpressed with SNRPB2 in ESCA. **(B)** KEGG pathway enrichment analyses of SNRPB2 correlated genes in ESCA. **(C, D)** GSEA enrichment analysis results of SNRPB2-related genes in ESCA. **(E)** The expression levels of genes related to the β-catenin/c-Myc signaling pathway were determined by western blotting. **(F)** Quantitative analysis of the changes in SNRPB2 protein levels. **P < 0.01, ***P < 0.001.

In order to delve deeper into the potential signaling pathways through which SNRPB2 promotes the progression of ESCA, SNRPB2-related gene analysis was conducted. A heatmap was generated to display the top 50 genes that exhibited the strongest correlation with SNRPB2 (**Figure 7A**). KEGG analyses were performed on 687 genes with a correlation coefficient greater than 0.3. The results indicated their involvement in pathways such as the Ribosome, Spliceosome, Oxidative phosphorylation, RNA degradation, and protein export (**Figure 7B**). Furthermore, GSEA enrichment analysis was conducted, revealing that SNRPB2 can activate pathways such as MYC targets pathway V1, E2F target, DNA repair, and Oxidative phosphorylation, while inhibiting pathways like p53 pathway and Hypoxia (**Figures 7C, D**). Abnormal expression of c-Myc is generally considered to be closely associated with cell proliferation, cycle, migration and invasion, while β-catenin is an important factor activating the expression of c-Myc in cancer cells. Additionally, β-catenin is also an upstream regulator for EMT (31). Therefore, we studied the correlation between SNRPB2 expression and β-catenin, c-Myc and the related cell cycle regulatory proteins. The experimental results showed that compared to the Sh-NC group, the knockdown of SNRPB2 significantly decreased the expression of β-catenin, c-Myc, CCNA2, CCNB1, CDK1, CDK2, while overexpression of SNRPB2 upregulated the expression of these genes (**Figures 7E, F**). These findings indicate that SNRPB2 can modulate the expression of proteins associated with the β-catenin/c-Myc signaling cascade.

### Stabilizing β-catenin restored the suppression of growth, migration, and invasion caused by SNRPB2 downregulation

**Figure 8:**
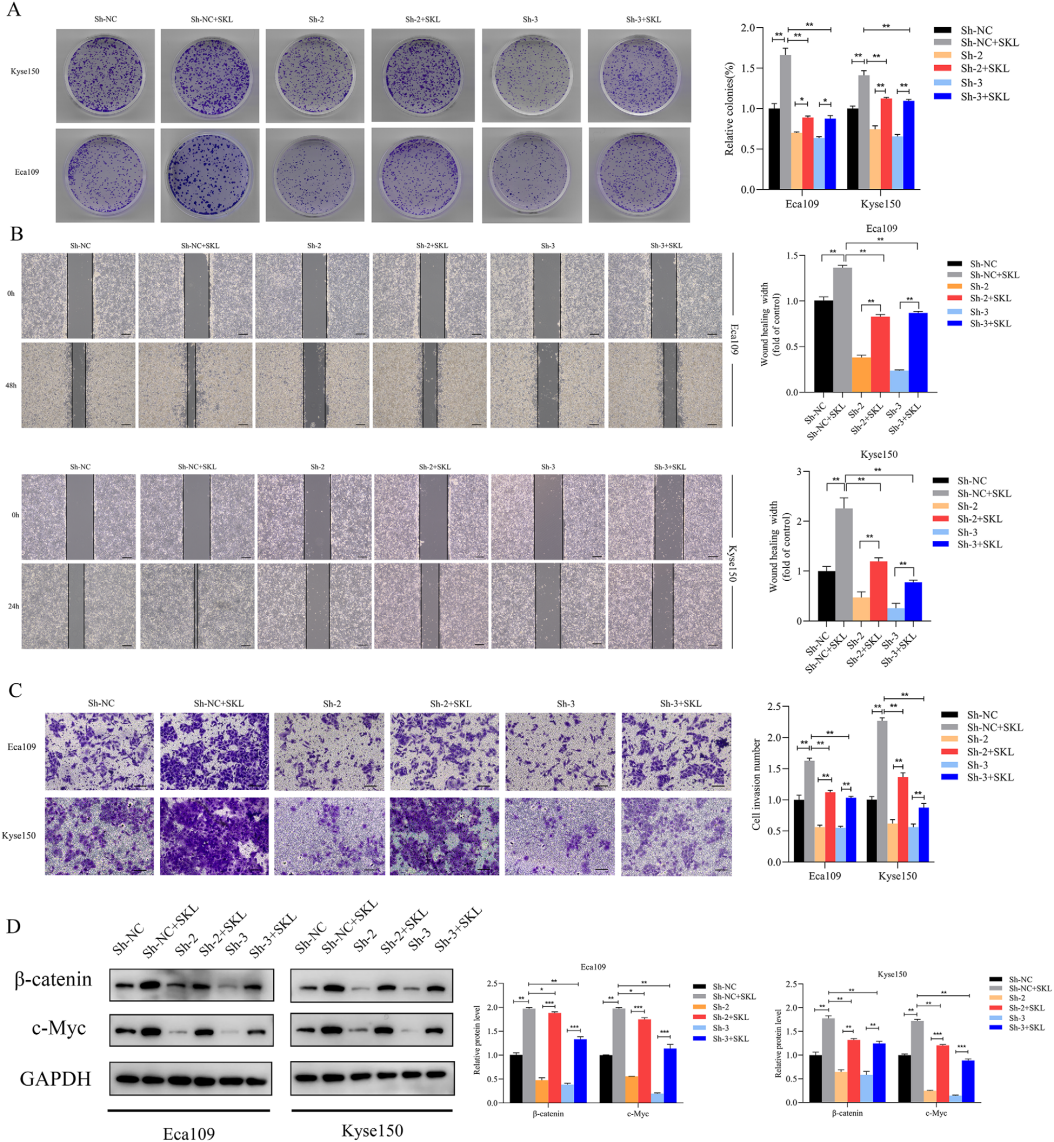
Stabilizing β-catenin restored the suppression of growth, Migration, invasion caused by SNRPB2 downregulation. **(A)** Colony formation assays showing the proliferation ability of Eca109 and Kyse150 cells infected with Sh-NC, Sh-2, and Sh-3 after 24 h treatment with SKL2001 (20 µmol/L). **(B)** Wound healing assays assessing the migration ability of Eca109 and Kyse150 cells under the same treatment conditions. **(C)** Transwell invasion assays demonstrating the invasive potential of Eca109 and Kyse150 cells. **(D)** Western blot analysis of β-catenin, c-Myc protein expression in treated cells. *P < 0.05; **P < 0.01.

Rescue experiments were conducted using the β-catenin agonist SKL2001 to investigate whether SNRPB2 influences ESCA cell proliferation, migration, and invasion via the β-catenin/c-Myc signaling pathway. Colony formation assays revealed that treatment with SKL2001 partially restored the reduced colony-forming capability associated with SNRPB2 knockdown (**Figure 8A**). Furthermore, after a 24-hour treatment with SKL2001, we harvested ESCA cells for migration and invasion assays. The results indicated that the SNRPB2 knockdown + SKL2001 group demonstrated significantly enhanced migration and invasion compared to the SNRPB2 knockdown group alone (P < 0.05) (**Figures 8B, C**). Following the 24-hour SKL2001 treatment, Proteins were collected from Eca109 and Kyse150 cells to assess the expression levels of β-catenin and c-Myc. As depicted in **Figure 8D**, SKL2001 effectively rescued the downregulation of both β-catenin and c-Myc that resulted from SNRPB2 knockdown.

### Inhibition of β-catenin restored the enhancement of growth, migration, invasion caused by SNRPB2 overexpression

**Figure 9:**
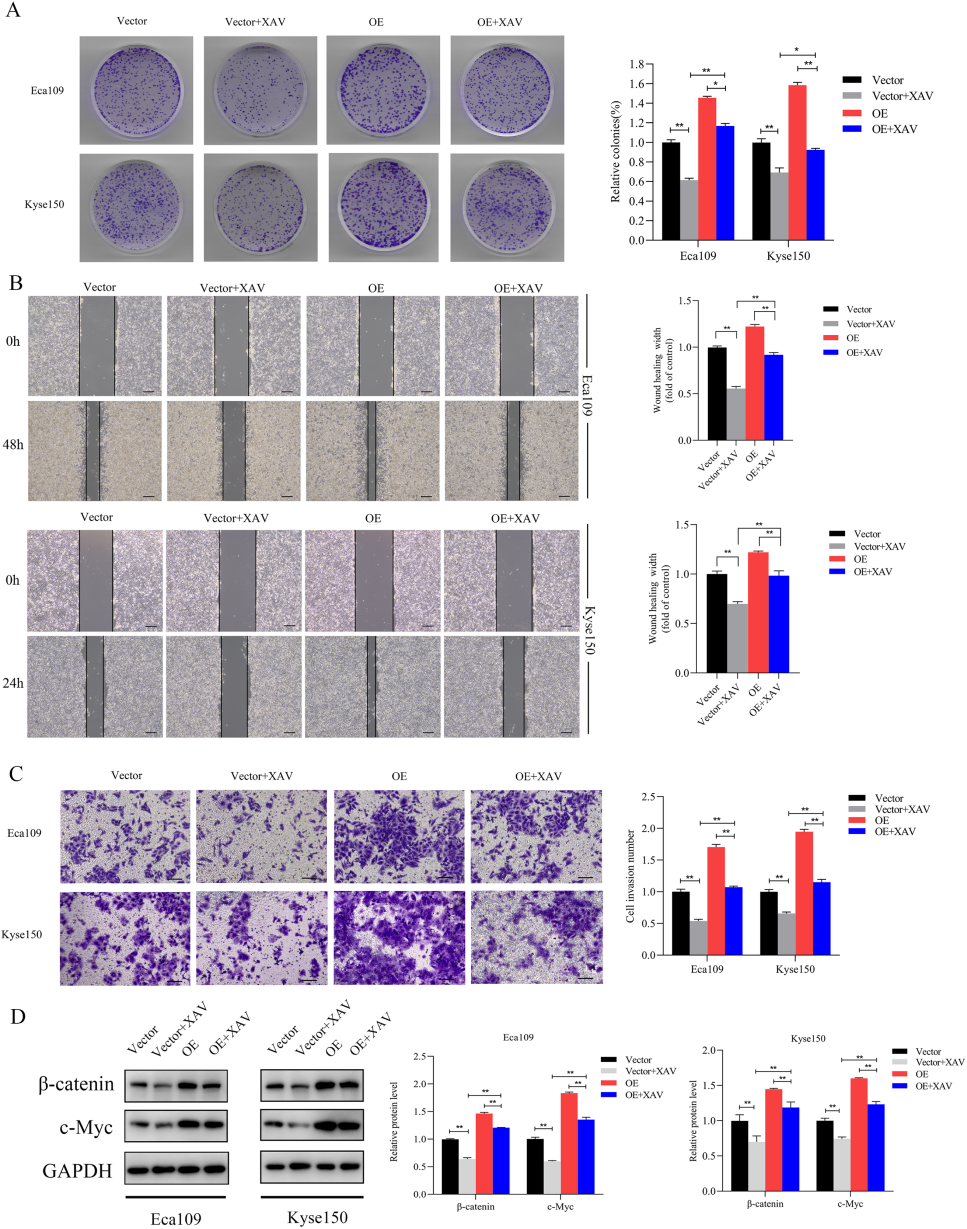
Inhibition of β-catenin reversed the growth, Migration and invasion promoted by SNRPB2 upregulation. **(A)** Colony formation assays showing the proliferation ability of Eca109 and Kyse150 cells infected with Vector, OE after 24h treatment with XAV-939 (20 µmol/L). **(B)** Wound healing assays assessing the migration ability of Eca109 and Kyse150 cells under the same treatment conditions. **(C)** Transwell invasion assays demonstrating the invasive potential of Eca109 and Kyse150 cells. **(D)** Western blot analysis of β-catenin, c-Myc protein expression in treated cells. *P < 0.05; **P < 0.01.

The β-catenin inhibitor XAV-939 was used to verify that SNRPB2 overexpression impacts ESCA cell proliferation, migration, and invasion via the β-catenin/c-Myc signaling pathway. Colony formation assays demonstrated that treatment with XAV-939 partially mitigated the increased colony-forming ability linked to SNRPB2 overexpression (**Figure 9A**). After exposing Eca109 and Kyse150 cells to XAV-939 for 24 hours, we conducted migration and invasion assays. The results indicated that in the SNRPB2 overexpression group treated with XAV-939, both migration and invasion abilities were significantly diminished compared to the group with SNRPB2 overexpression alone (P < 0.05) (**Figures 9B, C**). Proteins were collected from the Eca109 and Kyse150 cell lines following a 24-hour treatment with XAV-939 to assess the expression levels of β-catenin and c-Myc. As depicted in **Figure 9D**, the inhibitor effectively diminished the elevated expression of both β-catenin and c-Myc caused by SNRPB2 overexpression.

## Discussion

Alternative splicing (AS) emerges as a paramount post-transcriptional regulatory mechanism, exerting a pivotal role in augmenting protein complexity (32). According to statistics, over 95% of human genes are affected by AS, and aberrant AS functions are significant factors in tumor initiation (33). Multiple studies suggest a close association between the onset and progression of ESCA and AS (34–36). SNRPB2, a key component of the spliceosome, plays a crucial role in the process of alternative splicing. It was found early on that SNRPB2 is an early-growth response gene. Its enhanced expression may be upregulated in the transformation of oncogenes into fibroblasts during pre-mRNA conversion into mature mRNA, and it contributes to cell proliferation (37). Increasing evidence indicates that the expression of SNRPB2 is closely related to tumor initiation and progression. Dmitri V et al. reported that SNRPB2 participates in the downstream network activated by Invasion-promoting MT1-MMP, involved in cell reprogramming, transportation, cell division, energy metabolism, and other key cellular functions, ultimately leading to tumor occurrence (38). Yue Luo et al. demonstrated that lncRNA PCAT6 plays a key role in promoting the proliferation of hepatocellular carcinoma cells by regulating the cell cycle and apoptosis. Additionally, a significant correlation was identified between the expression levels of lncRNA PCAT6 and SNRPB2. Notably, SNRPB2 is markedly upregulated in hepatocellular carcinoma (26). A comprehensive pan-cancer exploration revealing SNRPB2 as a critical RNA splicing regulator with significant prognostic potential in cancer, and SNRPB2 silencing significantly suppresses proliferation and migration of Hepatocellular Carcinoma Cells (39). Yu-Tien Chang et al. identified SNRPB2 as a hub gene for distant metastasis of breast cancer through WGCNA and LASSO regression analysis (40). These findings further validate the potential of SNRPB2 as an oncogene, indicating that it plays a critical role in the occurrence and development of various tumors. However, the relationship between SNRPB2 and ESCA remains unclear.

Firstly, through bioinformatics analysis, we found that SNRPB2 is significantly overexpressed in the vast majority of tumors, including ESCA. Further combining database verification and immunohistochemical validation of clinical samples, we discovered that SNRPB2 is significantly upregulated in ESCA samples and is related to the stage progression of patients with ESCA. Our analysis also revealed that higher levels of SNRPB2 expression were linked to a poorer prognosis in patients with ESCA. The findings by Deng et al. indicate that SNRPB2 is upregulated in multiple myeloma, with patients showing low expression levels of SNRPB2 experiencing higher survival rates (25), which is consistent with our results. This implies that SNRPB2 could serve as a potential prognostic biomarker, which may help assess the prognosis of patients with ESCA and develop personalized treatment strategies.

To further confirm SNRPB2 as a promising therapeutic target for ESCA, we performed *in vitro* knockdown and overexpression experiments to observe its biological functions. Upon successful knockdown of SNRPB2, the growth rate, migratory ability, and invasive ability of ESCA cells were significantly inhibited. Conversely, overexpression of SNRPB2 significantly promoted these malignant phenotypes of ESCA cells. *In vivo* experiments further observed that after inhibiting SNRPB2, the tumor size was significantly reduced, and Ki-67 staining indicated that its proliferative ability was significantly weakened. EMT confers upon cells the ability to migrate and invade, induces stem cell traits, prevents apoptosis and senescence, and contributes to immune suppression (41, 42). Research has shown that silencing SNRPB2 in lung cancer cell lines, specifically H1299 and A549, leads to a decrease in the expression of EMT markers, including vimentin, MMP9, and MMP2 (43). Our research also revealed that the knockdown of SNRPB2 significantly reduces the expression of mesenchymal markers such as Vimentin and N-cadherin, while enhancing the expression of epithelial markers like E-cadherin and ZO-1, while overexpressing SNRPB2 can reverse this effect. These results indicate that SNRPB2 participates in regulating EMT, thereby influencing the progression of ESCA.

GSEA enrichment analysis revealed that SNRPB2-related genes activate the MYC target V1 signaling pathway. As a critical gene transcription regulator, c-Myc controls various key processes required for the development of many types of cancer, such as regulating cell cycle, cell competition, metabolism, tumor immune response, stem cell properties, and numerous other cellular activities (44). Multiple studies have indicated that β-catenin serves as a crucial activator in regulating the expression of c-Myc (45, 46). Wnt signaling is closely associated with the occurrence and progression of cancer (47). When the Wnt pathway is activated, the degradation of β-catenin is suppressed, leading to increased transport of β-catenin into the cell nucleus, where it interacts with downstream transcription factors of the LEF-1/TCF family. Subsequently, the activation of LEF-1/TCF facilitates the transcription of numerous genes, including c-Myc, c-jun, MMPs, and urokinase-type plasminogen activator receptor (48). In addition, the nuclear translocation of β-catenin also promotes the upregulation of N-cadherin and vimentin levels, while downregulating E-cadherin, in order to mediate epithelial-mesenchymal transition (EMT) during cancer metastasis (49). Chris Knill etal found that inhibition of the spliceosome component SNRPB during osteogenic differentiation leads to reduced WNT pathway activity and enhanced BMP pathway activity (50). Our results also confirm that SNRPB2 regulates the expression of β-catenin, thereby influencing the EMT process. As a key transcription factor, c-Myc is closely involved in the regulation of cell cycle-related proteins (46). c-Myc primarily stimulates the cell cycle by modulating cell cycle inhibitors (51). c-Myc promotes cell entry from G1 phase into the S phase, facilitating DNA synthesis. The complex formed by CCNA2 and CDK1 is essential for DNA replication (52). c-Myc also participates in driving cells from G2 phase to M phase. It can promote cell entry into mitosis by regulating the expression of Cyclin B1 and CDK2, as well as affecting the activation of M-phase promoting factor (MPF) (53). Our research findings indicate that inhibiting SNRPB2 affects the cell cycle progression of ESCA cells, particularly in the G2M phase. Moreover, the cell cycle-related proteins CCNB1, CCNA2, CDK1, and CDK2 were significantly downregulated. Conversely, the overexpression of SNRPB2 enhanced the levels of these cell cycle proteins, facilitating the transition of tumor cells from the G1 phase to the S phase and from the G2 phase to the M phase, thereby accelerating tumor cell proliferation.

The aforementioned research indicates that SNRPB2 enhances the levels of β-catenin, c-Myc, and various downstream proteins associated with proliferation, EMT and the cell cycle. Furthermore, the β-catenin activator SKL2001 effectively rescued the downregulation of β-catenin and c-Myc expression induced by SNRPB2 knockdown. In contrast, the β-catenin inhibitor XAV-939 counteracted the increased expression of β-catenin and c-Myc that was linked to SNRPB2 overexpression. Additionally, results from colony formation assays, wound healing experiments, migration, and invasion assays indicated that treatment with SKL2001 partially counteracted the effects of SNRPB2 silencing on cell proliferation, migration, and invasion. Conversely, treatment with XAV-939 partially mitigated the influence of SNRPB2 overexpression on these cellular processes. These findings imply that the regulatory mechanisms through which SNRPB2 influences proliferation, migration, invasion, EMT, and cell cycle transformation in ESCA may be partially mediated by the β-catenin/c-Myc signaling pathway. These findings underscore the oncogenic role of SNRPB2 and its potential as a therapeutic target for ESCA. Inhibition strategies could include small-molecule inhibitors or RNA-based approaches, such as siRNAs or antisense oligonucleotides, to suppress SNRPB2 expression. Targeting the downstream β-catenin/c-Myc axis with inhibitors like XAV-939 may further counteract its pro-tumor effects. Future studies should validate these approaches and assess their efficacy and safety, alone or in combination with conventional ESCA treatments.

However, there are certain limitations in our study. First, we relied on publicly available databases (TCGA, GEO, GTEX) to validate SNRPB2 expression and its prognostic significance; therefore, potential biases arising from batch effects or population heterogeneity cannot be ruled out. Second, the prognostic impact of SNRPB2 on ESCA requires confirmation through studies with larger clinical sample sizes. Finally, the interaction between SNRPB2 and β-catenin remains insufficiently explored, necessitating further research to clarify the underlying molecular mechanisms.

## Conclusions

Our study demonstrates that SNRPB2 is significantly overexpressed in ESCA and is closely linked to poor prognosis. SNRPB2 plays a critical role in promoting tumor proliferation, migration, invasion, and EMT, highlighting its contribution to the malignant characteristics of ESCA. Furthermore, its involvement in the β-catenin/c-Myc signaling pathway underscores its importance as both a prognostic biomarker and a potential therapeutic target. Targeting SNRPB2 and its associated pathways offers a promising strategy for controlling ESCA progression and developing more effective, personalized treatment approaches.

## Data Availability

The original contributions presented in the study are included in the article/supplementary material. Further inquiries can be directed to the corresponding authors.
